# Lesbian, gay, bisexual, transgender and gender diverse and queer (LGBTQ) community members' perspectives on palliative care in New South Wales (NSW), Australia

**DOI:** 10.1111/hsc.14024

**Published:** 2022-09-19

**Authors:** Nick J. Roberts, Lara A. Harvey, Roslyn G. Poulos, Éidín Ní Shé, Isabella Dillon Savage, Gemma Rafferty, Rebecca Ivers

**Affiliations:** ^1^ NSW Ministry of Health University of New South Wales Sydney New South Wales Australia; ^2^ Neuroscience Research Australia University of New South Wales Sydney New South Wales Australia; ^3^ University of New South Wales Sydney New South Wales Australia; ^4^ Royal College of Surgeons in Ireland Dublin Ireland; ^5^ NSW Ministry of Health St Leonards New South Wales Australia

## Abstract

Lesbian, gay, bisexual, transgender and gender diverse people, and queer people (LGBTQ people) are at increased risk of some chronic diseases and cancers. NSW Health palliative care health policy prioritises equitable access to quality care, however, little is known about community members' perspectives on palliative care. This study aimed to understand LGBTQ community views and preferences in palliative care in NSW. A community survey and follow‐up interviews with LGBTQ people in NSW were conducted in mid‐2020. A total of 419 people responded to the survey, with 222 completing it. Six semi‐structured phone interviews were conducted with participants who volunteered for follow‐up. The sample included LGBTQ people with varied levels of experience in palliative care. Thematic analysis was conducted on survey and interview data, to identify perceived barriers and enablers, and situate these factors in the socio‐ecological model of health. Some perceived barriers from community members related to considering whether to be ‘out’ (i.e., making one's sexual orientation and gender known to services), knowledge and attitudes of staff, concern about potential substandard care or mistreatment (particularly for transgender health), decision making, biological family as a source of tension, and loneliness and isolation. Perceived enablers related to developing and distributing inclusive palliative care information, engaging with community(ies), fostering inclusive and non‐discriminatory service delivery, ensuring respectful approaches to person‐centred care, and staff training on and awareness building of LGBTQ needs and issues. Most of the participants who had experienced palliative care recounted positive interactions, however, we identified that LGBTQ people require better access to knowledgeable and supportive services. Palliative care information should be inclusive and services respectful and welcoming. Particular consideration should be given to how services respond to and engage with people from diverse population groups. These insights can support ongoing policy and service development activities to further enhance palliative care.

**What is known about this topic?**

- Barriers to palliative care exist for the broader population and particular communities.
- LGBTQ people are at increased risk of certain chronic diseases that may require palliative care towards the end of life.
- Little is known about LGBTQ community members' opinions on palliative care in NSW, Australia.

**What this paper adds?**

- LGBTQ people in NSW who had experienced palliative care for themselves or someone close to them generally described respectful, inclusive interactions.
- Some community members expressed apprehension that wishes and preferences may not be met, often due to negative historical experiences
- The socio‐ecological model of health is useful for exploring palliative care with LGBTQ people

## 1 INTRODUCTION

Varied acronyms are used in this paper, acknowledging varied populations in the literature, and that questions in the current study tools related to lesbian, gay, bisexual, transgender and gender diverse and intersex (LGBTI) people, whereas respondents were from lesbian, gay, bisexual, transgender and gender diverse and queer (LGBTQ) communities.

The World Health Organisation describes palliative care as an approach to support patients and their families facing life‐threatening illness, through pain and other medical relief, and physical, psychosocial and spiritual support (2020). Service models vary globally (Bruera & Sweeney, 2002). In New South Wales (NSW), Australia, palliative care is delivered through various providers and settings, by multidisciplinary palliative care teams and specialists and other, non‐specialist health workers. These include general practitioners and nurses (NSW Government, 2019), community care workers (Poulos et al., 2018) and volunteers. Moving towards the end of one's life can be a period in which issues of sexuality, gender and embodiment may potentially be deprioritised for clinical concerns, including medication management and the settings of care and death (Griebling, 2016). However, sexual orientation and gender are relevant to considerations of the way in which palliative care can be accessed and delivered appropriately.

Various barriers exist to accessible and appropriate palliative care for the general population in Australia. These include timeliness of referrals to specialist palliative care (Johnson et al., 2020), community denial of death and dying (Zimmermann, 2007), effective communication between clinician and patient (Johnson et al., 2020) and people making their wishes for palliative care and end of life care known to their family and carers in advance (e.g., through advance care planning) (Scott et al., 2013). Some strategies to address barriers to access for diverse populations have been identified (Pentaris & Thomsen, 2018; Shahid et al., 2018).

Palliative care should be accessible for all population groups, including lesbian, gay, bisexual, trans (formerly described as transgender; Vincent, 2018), gender diverse, and queer people. LGBTQ people are at increased risk of some chronic diseases and cancers, which would be amenable to palliative care. For example breast and endometrial cancer for older lesbians (Cochran et al., 2001), and Hodgkin's disease, anal cancer and non‐Hodgkin's lymphoma (Smolinski & Colo'n, 2006) and HIV (NSW Health, 2022) for gay and bisexual men. Trans and gender diverse people are at increased risk of diabetes, cardiovascular disease and liver abnormalities (Williams & Freeman, 2007). Furthermore, the cohort of people in higher‐income countries who contracted HIV during the height of the epidemic are living longer and are more likely to require palliative care (Kirby Institute, 2018).

The literature identifies particular barriers to accessible and appropriate palliative care for people with diverse sexualities and sexual identities (Barrett & Wholihan, 2016; Bristowe et al., 2018; Marsack & Stephenson, 2018; Stevens & Abrahm, 2019), and that there are separate issues that need to be addressed for older LGBT people later years and end of life, in addition to those affecting the broader population (Almack et al., 2021). The international literature has addressed advance care planning (Cartwright et al., 2018; Dube et al., 2021; Marsack & Stephenson, 2018); LGBTQ people's experiences of palliative care (Bristowe et al., 2018; Kemery, 2021); clinical recommendations for palliative care with LGBT people (Maingi et al., 2018) and transgender people (Stevens & Abrahm, 2019); and education and training (Chidiac & Connolly, 2016; Sprik & Gentile, 2020), among other topics. Research on palliative care with these communities in NSW is limited, furthermore, the NSW research involving LGBT community members has focused on knowledge and perspectives of LGBT people on end of life and advance care planning alone, and not palliative care services (Cartwright et al., 2010; Hughes & Cartwright, 2014). The NSW research also predates key Australian socio‐political changes, including the legalisation of same sex marriage in 2018.

The socio‐ecological model (SEM) of health frames health as being influenced by an interaction between individuals, groups, environments and socio‐political contexts, among other factors (Ma et al., 2017), and it acknowledges interrelationships between factors (Stokols, 1996). The SEM has been applied widely to understand barriers and facilitators to care, including for healthy eating (Townsend, 2013) and breastfeeding obstacles (Bueno‐Gutierrez & Chantry, 2015). The model is useful to frame research relating to social justice, health, behavioural health and public policy (Henderson & Baffour, 2015). Davidson et al. articulated the SEM, in relation to palliative care and cultural diversity, theorising how ‘individual, provider and healthcare systems can influence how culture impacts on palliative care services and uptake’ (Davidson et al., 2016, p. 13).

Building on this articulation of the SEM, palliative care has also been examined through an SEM lens in a literature review relating to various sociodemographic groups (Nelson et al., 2021). To our knowledge, it has yet to be applied in studies about palliative care for LGBTQ people. In the current research, we considered that the SEM could also help in situating potential barriers and enablers to palliative care for LGBTQ people.

The aim of the study was to understand the perceptions of LGBTQ people in NSW on barriers and enablers to accessible and appropriate palliative care services.

## 2 METHODS

### 2.1 Overview

A mixed methods approach, involving an online community survey and qualitative interviews was undertaken (Mason, 2010). The study was approved by the University of NSW Human Research Ethics Committee (ref: HC200086). LGBTQ community organisational ethics approval was provided by the ACON Research Ethics Review Committee (ref: 2020/09).

Qualitative data were collected using an online community survey and follow‐up phone interviews were conducted with six community members. Community consultation was important for the survey planning, questionnaire development, pilot testing with 10 LGBTQ people and distribution. Pilot tests indicated the survey took 10–15 min to complete. The research team engaged with community organisations for feedback on the overall study aim, as well as draft questions.

### 2.2 Community survey

A self‐reported online survey was administered in June 2020 using Qualtrics, a web‐based platform. Participants were required to indicate online consent to participate and that they had read the participation information statement, prior to accessing the survey. The survey primarily contained open‐ended questions, to elicit opinions about palliative care service delivery. Two fictional examples of palliative care scenarios were provided to facilitate common understanding of what palliative care may involve, and the associated professionals (Appendix S1). Participants were recruited through social media posts by NSW Health and the LGBTQ community organisation, ACON. Invitations to participate were distributed to numerous LGBTIQ organisations. Inclusion and exclusion criteria are presented in Table 1.

**TABLE 1:** Inclusion/exclusion criteria (for survey and follow‐up interview participants)

Inclusion criteria	Self‐identify (through the survey) as: not heterosexual transgender or gender diverse, or having intersex variations
	Speak English
	Live in NSW, Australia
	18 years of age or older
	Willing to participate in the study and have provided informed consent
Exclusion criteria	Self‐identify as heterosexual, and not also transgender or gender diverse or having intersex variations

### 2.3 Qualitative follow‐up interviews with community members

Follow‐up phone interviews, lasting 35–45 min, were conducted in July–August 2020 with survey participants who had responded to an interview invitation at the end of the survey. Interviews were conducted by first author NR, a gay, cisgender male, senior policy officer, with experience conducting semi‐structured interviews. The follow‐up interview was semi‐structured (Patton, 2002), following a discussion guide with questions that varied, depending on whether the participant had experience of palliative care (for themselves, a partner or another LGBTI person close to them) or not (Appendix S2). For all interview participants, the interviews ended with the same demographic questions included in the community survey, to identify the level of diversity among this group. Audio recordings were made and transcribed by NR.

### 2.4 Thematic analysis

Survey and interview responses were analysed by NR. Partially complete surveys were included in the final analysis if at least the first open‐ended question had been answered (question B2 ‘What do you think could be some of the difficulties with using palliative care services for LGBTI people?’). This was to enable insights to inform the overall analysis from participants who may have exited the survey earlier, potentially due to survey fatigue or topic sensitivity. Descriptive statistics (Chi squared tests) were used to analyse the survey data from demographic questions, using Microsoft Excel (365 Pro Plus). Open‐ended responses were coded and analysed using an inductive thematic analysis approach (Terry et al., 2017). QSR NVivo (10) was used to collate and organise the de‐identified data and to assist with the application of themes and categorisation. Through the inductive approach, the research team discovered patterns, themes and categories in the data, by detailed interrogation and interaction with the data (Patton, 2002).

**FIGURE 1:**
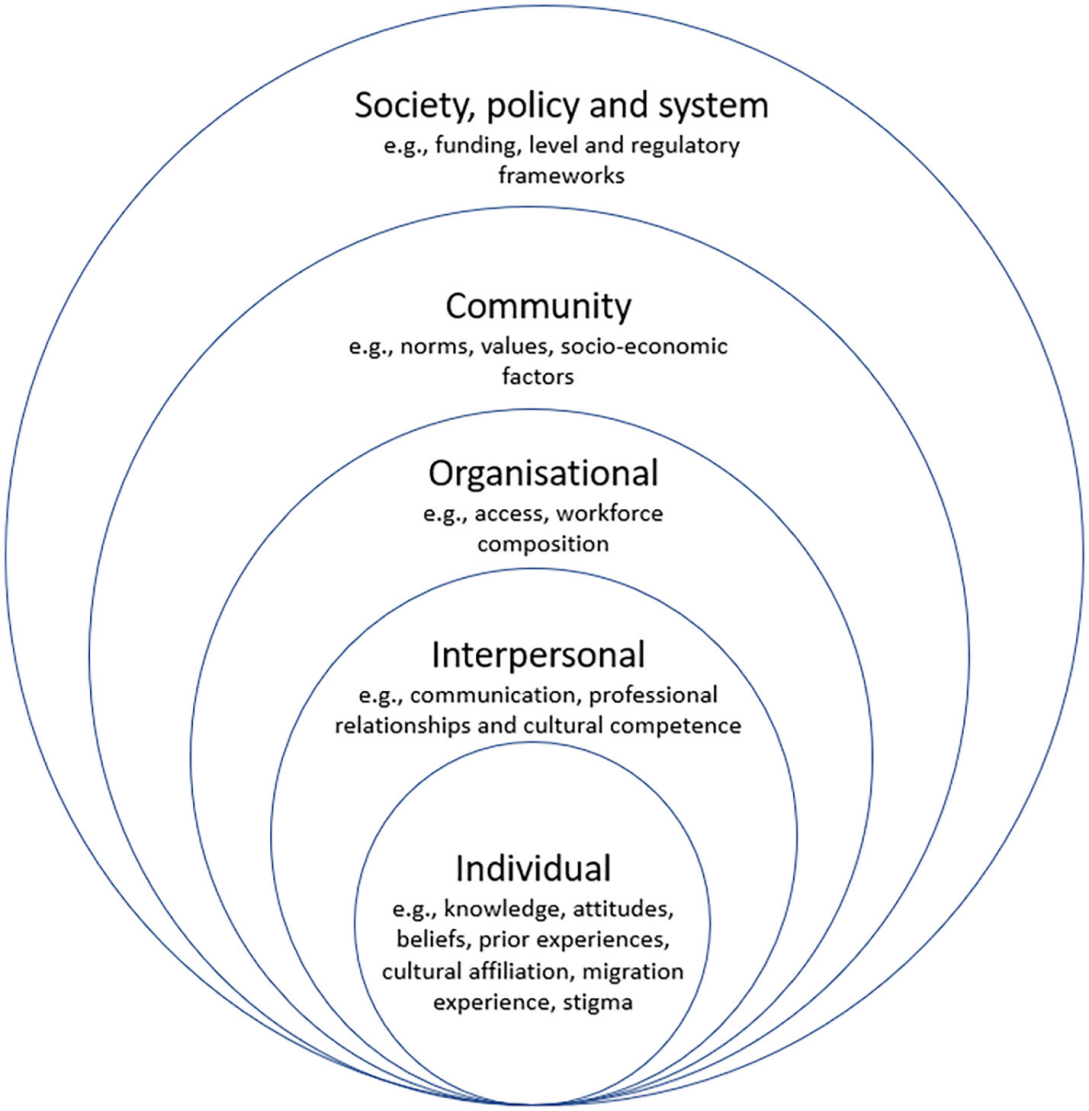
Social‐ecological framework for palliative care (Davidson et al., 2016, p. 13).

The themes were then applied to the SEM (Bronfenbrenner, 2009), adapted for the palliative care context by Davidson et al. (2016), using definitions provided by Davidson (Figure 1) as reference points for each level. It became apparent during this process that themes may relate to multiple SEM levels. In deciding on positioning of themes at the individual, interpersonal, organisational, community or society/policy/system level, NR considered the themes, in relation to anticipated patient experiences and service responses.

### 2.5 Rigour

The transcripts and survey data were re‐read and codes checked frequently to increase the reliability of the analysis (Flick, 2007). To help establish credibility (Lincoln, 1985), co‐author IDS coded a 10% sample of the survey responses and a 20% sample of the interview transcripts. NR and IDS then discussed similarities and differences in their codes (Austin & Sutton, 2015). This process resulted in some revisions to the codes and helped to clarify the final categories and themes. Survey and transcript data were combined to support triangulation during the interpretation stage.

NR regularly discussed the analysis and emerging themes with co‐authors, and through regular discussion determined the placement of the themes within the SEM. An audit trail supported dependability, details of the context supported transferability, and quotations are provided to support credibility.

## 3 FINDINGS

### 3.1 Participant characteristics

A total of 419 eligible participants started the survey with 222 answering the open‐ended questions (‘completed survey’). Six survey participants accepted the invitation to an interview, and all six were interviewed. Due to resource limitations for this research, further promotion and recruitment of additional interview participants did not take place. The characteristics of the 222 participants who completed the survey are similar to the full sample in all areas of the screening questions (Table 2). For sexual orientation: 54% of lesbian, bisexual and queer women (LBQW) and 53% of gay, bisexual and queer men (GBQM) completed the survey (*p* = 0.85). For gender: 54% of cisgender and 48% of transgender participants completed the survey (*p* = 0.74). For location, 52% of participants living in metropolitan NSW and 59% living in non‐metropolitan NSW completed the survey (*p* = 0.14). Of the 222 participants, 53 (24%) reported exposure to palliative care; 11 for themselves or a partner within the last 10 years, and 42 for another LGBTI person close to them.

**TABLE 2:** Participant characteristics (glossary of terms in Appendix S3)

	Survey respondents				Follow‐up interview participants	
	Total participants^a^ (*N* = 419)		Participants who completed at least one open ended question (*N* = 222)			
	*n*	%	*n*	%	*n*	%
Sexual orientation						
Lesbian	155	37.0	85	38.3	2	33
Gay	150	35.8	81	36.5	3	50
Bisexual+^b^	63	15.0	32	14.4	—	—
Same sex attracted, gender not specified	12	2.9	7	3.2	—	—
Queer	16	3.8	5	2.3	1	17
Heterosexual	3	0.7	1	0.5	—	—
Other orientations	4	1.0	2	0.9	—	—
Not specified	16	3.8	9	4.1	—	—
Gender						
Trans and gender diverse	46	11.0	22	9.9	—	—
Cisgender	371	88.5	199	89.6	6	100
Unspecified	2	0.5	1	0.5	—	—
Intersex status						
Intersex variation	4	1.0	2	0.9	—	—
Endosex	408	97.4	217	97.7	6	100
Prefer not to say	7	1.7	3	1.4	—	—
Geographic location						
Metropolitan NSW	314	74.9	162	73.0	4	67
Non‐metropolitan NSW	100	23.9	59	26.6	2	33
Prefer not to say	5	1.2	1	0.5	—	—
Age						
18–19	—	—	—	—	—	—
20–29	34	8.1	31	14.0		
30–39	51	12.2	47	21.2		
40–49	40	9.5	37	16.7		
50–59	43	10.3	41	18.5		
60–69	39	9.3	37	16.7		
70–79	21	5.0	20	9.0		
80+	3	0.7	3	1.4		
Prefer not to say	1	0.2	—	—		
Unspecified	187	44.6	6	2.7		
Cultural diversity						
Culturally and linguistically diverse^c^	41	9.8	32	14.4	1	17
Not culturally or linguistically diverse	183	43.7	184	82.9	5	83
Prefer not to say	8	1.9	0	0.0	0	0
Not specified	187	44.6	6	2.7	0	0
Living with disability?						
Yes	41	9.8	41	18.5	1	17
No	183	43.7	169	76.1	5	83
Prefer not to say	8	1.9	6	2.7	0	0
Not specified	187	44.6	6	2.7	0	0
Experience of palliative care^d^						
Respondent has personally used palliative care services	4		3		1	
Current or ex‐partner has used palliative care services within past 10 years	11		9		1	
Someone else close to respondent (who also identifies as LGBTI) has experience	48		48		0	
No experience of this	173		161		5	
Prefer not to say	3		2		0	
Not specified	186		6		0	

^a^ Following removal of spam responses; people identifying as heterosexual, cisgender and endosex. Offensive, ineligible or otherwise invalid responses also removed.

^b^ ‘Bisexual+’ has been used to refer to participants identifying their sexual orientation as pansexual or panromantic.

^c^ Based on AIHW/ABS publications, categorised as culturally and linguistically diverse if person either spoke language other than English at home, or they were born overseas in a non‐main English‐speaking country (e.g. not United States, United Kingdom, New Zealand, Ireland).

^d^ Some participants disclosed multiple forms of experience (e.g., for self and others).

### 3.2 Positive experiences of palliative care

Various positive aspects of care were recounted by the 53 participants reporting exposure to palliative care. Common experiences included palliative care services supporting the patient in a manner of dignity and respect. This respect included support for their chosen family, and incorporating partner and/or their chosen family in the care. Correct gendering and use of the preferred name for the patient were particularly appreciated. Facilities with a long history of working with the LGBTQ community were valued.

### 3.3 Barriers

Six categories of barriers were identified. The first three barriers relate more specifically to palliative care services, whereas the last three are general concerns. These perceived barriers were identified by both participants who had, and had not, experienced palliative care. Further quotes are presented in Table 3a. Barriers identified through the survey data, by sexual orientation, gender and location are presented in Table 4a.

**TABLE 3:** Participant quotes (a) barrier and (b) enablers

	Participant number	Participant characteristics (sexual orientation, gender, location in NSW)	Quote
(a) Barriers			
Considering whether to be out to service providers or not	S190	Gay, Cisgender, Metro	LGBTI people being concerned that they would have to ‘de‐gay’ or ‘de‐queer’ their lived‐in environment to avoid possible judgement of who they are or their life‐style
	I1	Lesbian, Cisgender, Metro	Some people prefer not to talk about it. They may even feel uncomfortable for others to know about it
Care Staff and Services	S42	Gay, Cisgender, Non‐Metro	If the people providing the care are not used to dealing with queer people they may react differently to a loving kiss or two men holding hands, etc., and I could imagine that would be really distressing if it happened in your house
	S66	Lesbian, Cisgender, Non‐Metro	A lack of understanding as to what we define as family. It is a different concept to traditional family models that most staff would understand
	S44	Pansexual, Trans man, Metro	Judgement, care teams not understanding the needs of patients, or ignorance about respectful terminology (=misgendering, assumption about sexuality or slurs)
	S309	Bisexual, Cisgender Female, Metro	Fear of judgement. I know that a lot of palliative care is provided by the Catholic church and other faith‐based groups who have been very vocal in their prejudice against LGBTI people
	S159	Lesbian, Cisgender, Metro	My experience of palliative care has been that individual staff members make all the difference and as long as they are compassionate and caring of LGBTI and partners, friends and family as they are with anyone else it is fine
	S12	Queer, Cisgender, Non‐Metro	Metropolitan areas (have) more specialised services and resources than rural/regional areas in the first place which can mean rural living LGBT people have to move away from their families/homes to get affirming care that meets their needs—including palliative … LGBT people who remain in smaller rural communities can be subjected to greater instances of discrimination/conservatism
Concern about potential substandard care or mistreatment	S149	Bisexual, Non‐Binary/gender fluid, Non‐metro	Some of us have very very bad experiences of the medical profession *…* e.g. forced hormonal treatment *…* forced mental health lock ups … and even more serious abuses *…* such as sterilisation … carers need to know about this and understand the impact it has including flashbacks when dealing with medical providers*…*
	S86	Bisexual, Cisgender, Non‐Metro	A lot of older LGBTI people have experienced horrendous discrimination and violence at the hands of institutions (e.g. police, hospitals, psychiatric facilities, etc.) and have witnessed friends and loved ones being discriminated at the end of their lives. For example, families shutting out partners at the bedside, and people who have lived through the AIDS epidemic and suffer survivor guilt
Biological family issues as a source of tension	S21	Pansexual Female, Cisgender, Metro	These family members may not be supportive of the palliative person's orientation, choices, partner, gender identity, etc., and bar any same sex (partners) from visiting, etc.
	S149	Bisexual, Non‐Binary/Gender Fluid, Non‐Metro	*…* sometimes extended family still do not feel that the same sex partner is really part of the family and/or has a say in (the) medical decision …. palliative care workers in particular need to be aware of this and ensure the wishes and rights of a same sex couple are not derailed by unhappy extended family … otherwise for some LGBTI people … family is more fluid and strong ties between other individuals and the patient need to be acknowledged and supported …
Next of kin and decision making	S309	Bisexual, Cisgender Woman, Metro	When it comes to dying, the family issues can be very difficult to navigate especially determining next of kin. For example, disputes about who has the right to act as a substitute decision maker if the patient loses capacity. Will same sex partners be recognised?
	I3	Gay, Cisgender, Metro	this question would've been extremely important a few years back when the marriage, the gay, LGBTI marriage was not valid, but considering it is legal here to get married to a same sex partner and also considering that Australia has the de‐facto relationship, I would assume the person who is in a de‐facto relationship would have the same authority in a situation where the main person cannot make a decision, and I'm sure the rules cater to that
Loneliness and isolation	S176	Gay, Cisgender, Metro	Many gay people live alone and do not have a partner to support them. Their friends (often of the same age cohort) are not necessarily willing or able to assist when someone is in the terminal stages of their illness
	I4	Lesbian, Cisgender, Non‐Metro	…a friend of mine, her mother was cared for at home, right through her palliative care, she passed away at home. And it was done very well. There was a palliative care nurse that came in. To start with it was like once a week, and once it progressed, it was every day, sort of thing. To try and help the family through. But lots of times, people in our community have no family. If their partner's gone and we are of an age 70*…* maybe all of your friends are passed away or they are doing their own thing. So really, sometimes it's just you and your dog (laughs)
(b) Enablers			
Developing and distributing inclusive information about palliative care and services	S168	(sexuality not specified) Non‐Binary Trans Masculine, Metro	To have positive stories told, that death is discussed, embracing Indigenous dream time. I think that older LGBTQI people are on the frontier of experiencing (death and dying), so recording that in a positive light, and to create a separate support LGBTQI friendly carer groups
	S260	Lesbian, Cisgender, Metro	Have some same sex/trans/gender fluid people in posters/brochures … make inclusivity the norm*…* Have information in medical spaces that LGBTI people use
	S112	Same sex attracted, Non‐Binary/Gender Fluid, Metro	Provide information in queer friendly spaces—particularly physical spaces such as medical clinics, community organisations or volunteer groups, etc.
	S42	Gay, Cisgender, Non‐Metro	Advertise in media that we might follow *…* you could partner with one of those publications to do a section on queer health around the end of life. I think that palliative stirs up negative connotations because the only other time you hear a word like that is ‘pall bearers’ at a funeral. If it's something that does not signify immediate death (which I guess is the misconception) then I think you should try and educate the population about it's true meaning and how it is an integral and important part of any health care system
Engaging with the LGBTQ community(ies)	S31	Queer, Non‐Binary/Gender Fluid, Metro	Engage with community organisations (e.g. PosLife, ACON) and ensure discussion with consumers
	S15	Gay, Cisgender, Metro	Have a stall at Fair Day, float in Mardi Gras. Present at local and regional prides. Work with existing charities *…* to let them know services are there
	S15	Gay, Cisgender, Metro	Have a stall at Fair Day, float in Mardi Gras. Present at local and regional prides. Work with existing charities *…* to let them know services are there
Fostering inclusive and non‐discriminatory service delivery	S16	Gay, Cisgender, Metro	Advertise inclusivity and diversity, e.g. (staff) who have completed special training wearing an identifiable pin or a mention in their title
	S44	Pansexual, Trans Man, Metro	Clear markings showing that the organisation is LGBTQIA+ friendly (pride flag, posters, extensive gender/sexuality options on forms). Assurance that staff has been trained on LGBTQIA+ etiquette and medical needs (where necessary)
Improving understanding of palliative care among LGBTQ people	S184	Gay, Cisgender, Non‐Metro	Community engagement for end‐of‐life conversations and the options that are available while you are living well—early open discussions. Try a community ‘Death café’ to start the death and dying yarns
	S311	Bisexual, Cisgender, Metro	Education re palliative care likely need not differ to other communities, but rather making it something that LGBTI community does not need to fear being judged
Staff approach to person‐centred care	S149	Bisexual, non‐binary/gender fluid, non‐metro	be matter of fact … do not make a big deal of the sexuality while relating to any partners and loved others warmly … a tricky balancing act sometimes …
	S243	Pansexual, Trans man, Non‐Metro	Let LGBT+ people know how your palliative care is going to be specific to their needs
	I2	Gay, Cisgender, Non‐Metro	because I do not drive, they actually let me stay and sleep next to (partner, undergoing palliative care) on one of those care chairs or something, and so I could stay a night or two. I did not even have to hint, I think (my partner) may have asked a couple of times. But as time went on, I just felt welcome on that level too. So, it's important to make a person's partner feel welcome and part of the process
	S346	(sexuality not specified) Trans woman, Metro	(Do) not positively discriminate. Just allow us to be part of society without trying to over accommodate our difference in society
Staff training and awareness in LGBTQ needs and issues	S45	Queer, non‐binary/gender fluid, metro)	Make sure there have been updated training programmes (at the service). Also, assure the LGBTQIA+ people that everyone in the (service) is on board and accepting. Have a criteria for people who work (in the service). If they have a problem with working with LGBTQIA+ people, they do not have the privilege to work there
	S42	Gay, Cisgender, Non‐Metro	*…* there would be clearly advertised (palliative care) services with specialist training for the carers in what to expect. The only thing different about my house is that 2 guys share it, but we know from past experience that can be confronting for some people (workers)
	S142	Lesbian, Trans woman, Metro	People need to understand about LGBT and have no problems with LGBT

*Note*: Participant number prefix S denotes survey participant; prefix I denotes follow‐up interview participant.

**TABLE 4:** Barriers and enablers (from survey data)

	Sexual orientation (lesbian, & bisexual+ & queer women—LBQW; gay, & bisexual+ & queer men—GBQM)						Gender (Trans and gender diverse—TGD; cisgender—Cis)				Location				Total (*n* = 222)	
	LBQW (*n* = 109)	%	GBQM (*n* = 86)	%	Bisexual+ (*n* = 32)	%	TGD (*n* = 22)	%	Cis (*n* = 199)	%	Metro NSW (*n* = 162)	%	Non‐metro NSW (*n* = 59)	%	*N*	%
(a) Barriers																
Being out at services or not	11	10	5	6	4	13	1	5	16	8	11	7	6	10	17	8
Biological family issues	10	9	11	13	4	13	2	9	22	11	19	12	5	8	24	11
Care staff and services	89	82	62	72	28	88	19	86	155	78	124	77	49	83	174	78
Concern of substandard care or mistreatment	20	18	11	13	14	44	9	41	31	16	29	18	12	20	41	18
Next of kin and decision making	11	10	9	10	6	19	3	14	21	11	18	11	6	10	24	11
Loneliness and isolation	2	2	8	9	0	0	0	0	10	5	9	6	1	2	10	5
(b) Enablers																
Developing and distributing inclusive info and marketing	52	48	52	60	14	44	10	45	106	53	79	49	37	63	116	52
Engage with LGBTQ community	40	37	27	31	14	44	13	59	68	34	59	36	22	37	81	36
Foster inclusive and non‐discriminatory service delivery	64	59	44	51	16	50	12	55	107	54	89	55	30	51	119	54
Improve understanding of palliative care among LGBTQ people	14	13	8	9	8	25	1	5	26	13	18	11	9	15	27	12
Staff approach to person‐centred care	54	50	30	35	19	59	11	50	86	43	67	41	30	51	97	44
Staff training and awareness in LGBTQ needs and issues	35	32	19	22	14	44	7	32	55	28	41	25	22	37	63	28

#### 3.3.1 Barrier 1—Considering whether to be ‘out’ to service providers or not

Participants reported concern that patients may conceal their sexuality or gender to palliative care service providers, for fear of possible judgement, discrimination or their identity being disclosed by the service providers to their families:

…LGBTI people being concerned that they would have to ‘de‐gay’ or ‘de‐queer’ their lived‐in environment to avoid possible judgement. [gay, cisgender, metro]

They noted that some patients, particularly older people may feel less comfortable disclosing their identity, and expressed concern about the fatigue that people can feel from having to repeatedly come out (disclose their identity or embodiment).

#### 3.3.2 Barrier 2—Care Staff and Services

Concerns were raised regarding perceived knowledge and attitudes of people working in services delivering palliative care towards LGBTQ people receiving care:

A lack of understanding as to what we define as family. It is a different concept to traditional family models that most staff would understand. [lesbian, cisgender, non‐metro]

These concerns primarily related to staff of the service and negative views they may hold towards LGBTQ people. This was considered by community members as likely to impact on a patient's level of comfort accessing palliative care services. The worries included potential attitudes towards the person's chosen family or their partner and non‐traditional relationships more broadly and the perceived heteronormativity of ‘mainstream’ services (i.e. taking a world view that promotes heterosexuality as the normal or preferred sexual orientation). Worries also included the potential for staff to exhibit phobia (i.e., homophobia, bi‐phobia or transphobia), prejudice, stigma or discrimination.

A strong sub‐theme was that staff could lack understanding of LGBTQ people, their lives and health needs, with several people raising concern that several facilities are faith‐based. They felt that this may manifest in potential negativity towards LGBTQ people.

Despite concerns about potential level of staff knowledge and attitudes, a majority of the participants who had experienced palliative care for themselves or another LGBTQ person close to them (survey *n* = 53; interview *n* = 1) recounted positive experiences.

Some examples of potential service‐level barriers to palliative care for people living outside of cities were provided. Perceived conservatism outside of cities was also highlighted, and the impact this may potentially have on engagement with services.

#### 3.3.3 Barrier 3—Concern about potential substandard care or mistreatment

Some participants felt that LGBTQ people may be subject to a lower standard of care from services, or that the service (and staff) may be unresponsive to their needs. At the extreme, a few participants held concerns that LGBTQ people could potentially be subject to mistreatment. Concerns tended to stem from the traumatic or otherwise negative historical experiences of health services endured by community members, particularly elders:

Some of us have very, very bad experiences of the medical profession … e.g., forced hormonal treatment … forced mental health lock ups … carers need to know about this and understand the impact it has …[bisexual, non‐binary, non‐metro]

Participants recognised this manifesting in a distrust of health services in general. Concerns for trans and gender diverse people were that they may be misgendered or not able to maintain access to gender‐affirming medications while receiving palliative care.

#### 3.3.4 Barrier 4—Next of kin and decision making

Several participants raised decision making concerns, in particular the potential for patient wishes for next of kin or person responsible to not be recognised. If these wishes are not recognised, participants identified the potential for disagreements between the patient's biological family and their chosen family and partner:

disputes about who has the right to act as a substitute decision maker if the patient loses capacity. Will same sex partners be recognised? [bisexual, cisgender woman, metro]

However, the introduction of marriage equality to Australia in 2017 was understood to help ‘validate’ wishes around next of kin, in this regard, although there was still some lack of understanding and confusion around legal status.

#### 3.3.5 Barrier 5—Biological family issues as a source of tension

In addition to decision making, other perceived challenges relating to biological family were raised. These include estrangement from a patient's biological family, patients not being out to their biological family; patient decisions on reconciling (or not) with family members in their last days of life; and the level of support and acceptance (or otherwise) of the biological family towards the patient and/or their partner:

These family members may not be supportive of the palliative person's orientation, choices, partner, gender identity… [pansexual female, cisgender, metro]

#### 3.3.6 Barrier 6—Loneliness and isolation

Some participants identified societal factors, including loneliness and social isolation experienced by some LGBTQ people. There was concern that LGBTQ people may experience loneliness and could be more socially isolated, because of being less likely to have a surrounding biological family, and potential negative attitudes based on their identity expressed by the broader society:

But lots of times, people in our community have no family. If their partner's gone and we are of an age 70… maybe all of your friends are passed away. [lesbian, cisgender, non‐metro]

### 3.4 Enablers

Six categories of enablers of access to, and engagement with, palliative care were raised. Further participant quotes are presented in Table 3b.  Enablers identified through the survey data, by sexual orientation, gender and location are presented in Table 4b.

#### 3.4.1 Enabler 1—Developing and distributing inclusive information about palliative care and services

It was suggested that materials should represent the diversity of the community(ies) and include palliative care information as well as advertising for the services available. Suggestions were made for targeted materials and that photos of LGBTQ people should be featured in mainstream (non‐targeted) materials:

Have some same sex/ trans/ gender fluid people in posters/brochures …make inclusivity the norm… [lesbian, cisgender, metro]

Suggested channels to distribute materials included social media, websites, health services and clinics accessed by community members and through community ‘champions’ of palliative care.

#### 3.4.2 Enabler 2—Engaging with the LGBTQ community(ies)

It was felt that services could engage with the community(ies), whilst acknowledging diversity of the community(ies). It was suggested that services engage with organisations and services that currently support the community(ies):

Have a stall at Fair Day, float in Mardi Gras. Present at local and regional prides. Work with existing charities… [gay, cisgender, metro]

This process was seen as key to building trust in services, disseminating information and building capacity, knowledge and understanding of services.

#### 3.4.3 Enabler 3—Fostering inclusive and non‐discriminatory service delivery

Approaches to enhance the delivery of palliative care services were raised. These included displays of inclusion within service premises:

Clear markings showing that the organisation is LGBTQIA+ friendly (pride flag, posters, extensive gender/sexuality options on forms). [pansexual, trans man, metro]

Suggestions also included employing LGBTQ staff in identified (i.e., where the employer identifies that a position is to be filled only by a person who identifies as LGBTQ [Australian Human Rights Commission, 2015]) and non‐identified positions. Further suggestions included improving the understanding of LGBTQ people among palliative care workers, ensuring there is no bias or discrimination in service delivery, and reviewing service policies, practices and accreditation status.

#### 3.4.4 Enabler 4—Improving understanding of palliative care among LGBTQ people

Suggestions were made to foster and improve the palliative care (and death) literacy of community members. These related to community events and education sessions, that may de‐stigmatise death and palliative care.

It was also suggested that education could (or should) take place in the same manner as for the wider population:

Education re palliative care likely need not differ to other communities, but rather making it something that LGBTI community does not need to fear being judged. [bisexual, cisgender, metro]

#### 3.4.5 Enabler 5—Staff approach to person‐centred care

An approach of respect, acceptance, affirmation and support towards LGBTQ people was emphasised. Suggestions included management addressing any problematic behaviour within the service towards LGBTQ people; staff advocacy for patients and LGBTQ issues; asking patient pronouns and correctly gendering trans and gender diverse people; open discussions of care with patients and recognising and supporting partners and chosen family:

…they actually let me stay and sleep next to (partner, undergoing palliative care) … it's important to make a person's partner feel welcome and part of the process. [gay, cisgender, non‐metro]

An alternative viewpoint, cautioning against palliative care services ‘over‐attending’ to these needs was also raised, as was the importance of addressing requirements of multicultural LGBTQ people.

… Just allow us to be part of society without trying to over‐accommodate our difference in society. [trans woman (sexuality unspecified), metro]

#### 3.4.6 Enabler 6—staff training and awareness in LGBTQ needs and issues

Education and training to improve awareness and understanding of LGBTQ people among palliative care specialist and non‐specialist workers were suggested.

Make sure there have been updated training programmes (at the service) *…* Have a criteria for people who work (in the service). [queer, non‐binary, metro]

Participants suggested such training for non‐specialist workers should include GPs.

**FIGURE 2:**
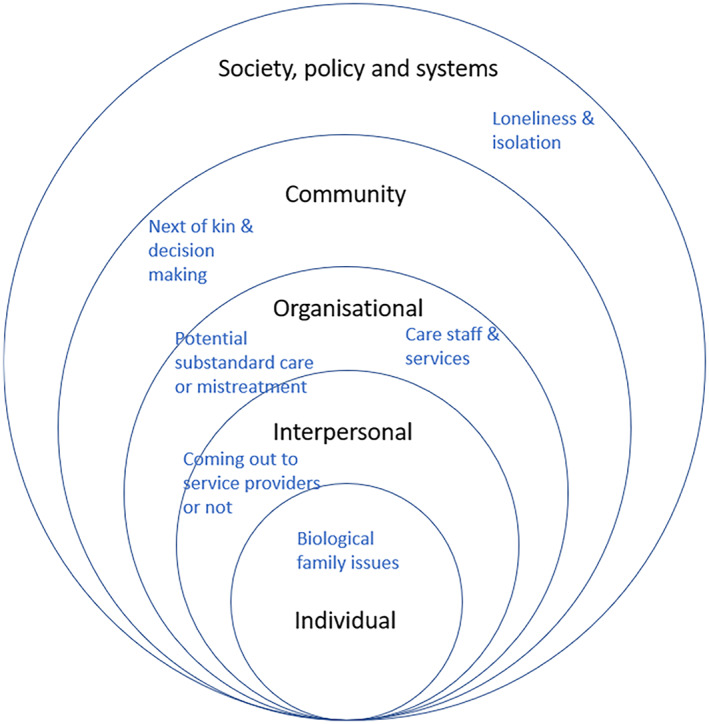
Potential barriers perceived by community members (for all participants).

**FIGURE 3:**
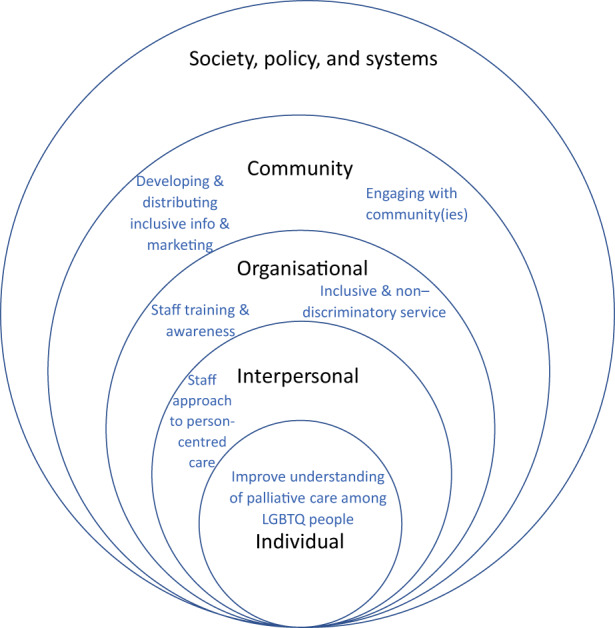
Potential enablers perceived by community members (for all participants).

The potential barriers and enablers to accessible and appropriate care, and their alignment to the levels of the SEM (Davidson et al., 2016) are shown in Figures 2 and 3. Table 5 includes commentary on positioning at the SEM levels.

**TABLE 5:** Commentary on positioning of themes at levels within the SEM

Level within the SEM	Theme	Commentary
*Barriers*		
Individual level	Biological family issues as a source of tension	Relating to historical and current experiences and dynamics of LGBTQ individuals, with respect to their biological families
Interpersonal level	Considering whether to be out to service providers or not	Decisions of LGBTQ people to disclose their sexual orientation or gender to service providers may be influenced by (and may influence) interactions between patients and service providers
Organisational level	Care Staff and Services	Relates to features centred around the service, including workforce, policies, procedures and other aspects that may impact on access or appropriateness of care
	Concern about potential substandard care or mistreatment	
Community level	Next of kin and decision making	LGBTQ peoples' decisions for next of kin will be influenced by their relationships with partners, biological and chosen family and other members of their communities
Society, policy and systems level	Loneliness and isolation	Loneliness and isolation are social experiences which relate to a persons' situation (or perceived) situation in relation to the broader society

Level	Theme	Commentary
*Enablers*		
Individual level	Improving understanding of palliative care among LGBTQ people	Davidson notes that knowledge, attitudes and beliefs are moderated by sociocultural factors, peer experiences and prior contact with palliative care services (2016)
Interpersonal level	Staff approach to person‐centred care	The relationships formed between staff members, patients and their family or carers are supported through respectful, affirmative communication, which can facilitate discussions about values and preferences for palliative care
Organisational level	Staff training and awareness in LGBTQ needs and issues	Through enhancing the skills, knowledge and capacity of staff, organisations can support the workforce to understand the needs LGBTQ community members may experience, and how they may respond. Inclusive and non‐discriminatory service delivery is pivotal to accessible and appropriate care, including gaining and maintaining the trust of community members
	Fostering inclusive and non‐discriminatory service delivery	
Community level	Developing and distributing inclusive information about palliative care and services	Engagement with LGBTQ community(ies) can facilitate the development and distribution of inclusive information to said communities. This engagement can help organisation to navigate community ‘gateways’ and communicate in an appropriate and engaging manner with the LGBTQ community(ies)
	Engaging with the LGBTQ community(ies)	
Society, policy and systems level	(no themes identified in the data)	

## 4 DISCUSSION

This paper has described the views on palliative care of a selection of people with diverse sexualities and gender identities who live in NSW, Australia. It reveals for the first time their concerns, hopes and suggestions around how palliative care services can be made more appropriate and accessible for their community(ies). It also presents a novel application of the SEM (Davidson et al., 2016) in presenting barriers and enablers to palliative care for these community(ies). This framework can help services to recognise where they may be able to intervene or make changes to provide more accessible and appropriate services for the community(ies). This research builds upon the existing literature on how to make palliative care services more inclusive for LGBTQ people (Acquaviva 2017; Lintott et al., 2022) and brings increased understanding of LGBTQ people's experiences of palliative care, a topic that has received little attention in the literature.

The themes are discussed, in relation to levels in the SEM.

### 4.1 Individual level barriers and enablers

Participants expressed potential concerns relating to biological family issues as a source of tension, including potential estrangement and consequential impacts on decision making. Previous research on NSW LGBT community members' knowledge and perspectives on end of life and advance care planning has relevance here (Cartwright et al., 2010; Hughes & Cartwright, 2014). Cartwright et al., 2010 found 9% of community members were not open to any significant family members about their sexuality, noting that people who do not disclose their sexuality may be at particular risk in relation to their end of life care.

Participants suggested that the understanding of community members of palliative care could be improved, to facilitate palliative care and death literacy. Whilst suggestions were made on implementing this through inclusive materials and community engagement, participants saw this as a component of improving the understanding within the broader community. National policy includes an emphasis on reducing stigma around death and dying (Department of Health, 2018) and it is important that death literacy activities are inclusive and affirming of LGBTQ people.

### 4.2 Interpersonal level barriers and enablers

Considerations of whether participants should come out to service providers or not were raised as a potential barrier to engagement with palliative care services. The survey introduction emphasised the multi‐disciplinary nature of palliative care teams, and this may have caused some participants to reflect on the need to repeatedly come out to various service providers. Wilson, et al. (2018) have noted the ‘fear and dread’ that many LGBT people in Canada feel towards the prospect of having to return to the closet towards the end of life, to receive quality care. Anticipation of inappropriate care or lack of cultural competence (Aldredge & Conlon, 2012; Bristowe et al., 2016, 2018; Cartwright, 2012; Griebling, 2016; Stevens & Abrahm, 2019; Stinchcombe et al., 2017) has also been identified. Such concerns highlight a need for services to reassure community members that they will receive appropriate and high‐quality treatment by the service provider, regardless of whether the person has chosen to disclose their identity to staff members or otherwise.

The staff approach to person‐centred care, including the way staff relate to patients, their families and carers, was identified as an important strategy at the interpersonal level. Lesbian, bisexual and queer women (and bisexual people overall) often suggested that the staff approach to person‐centred care would be an enabler to palliative care (see Table 4b). Respect, acceptance and support towards LGBTQ people were emphasised, without staff making a ‘big deal’ about patient's identity in their interactions. Language use (for example, correctly gendering people by using their correct pronouns and names) is highly important to help engage and earn the trust of trans and gender diverse people (Pitts et al., 2009), and should be respected.

### 4.3 Organisational level barriers enablers

Some concerns were raised about the knowledge and attitudes that staff of services delivering palliative care may hold towards LGBTQ people, and the potential discomfort this may elicit in patients, their partners and chosen families. Some unease was expressed about the perceived heteronormativity of ‘mainstream’ services, and potential for stigma or discrimination. Previous literature has uncovered concerns about end of life and palliative care, relating to discrimination against LGBTI people by services (actual, perceived or anticipated) and/or heteronormativity (i.e., a world view that promotes heterosexuality as the normal or preferred sexual orientation) (Barrett & Wholihan, 2016; Cartwright, 2012; Chidiac & Connolly, 2016; Marsack & Stephenson, 2018; Stinchcombe et al., 2017).

At the more extreme end, the apprehension some people have about receiving a lower standard of care from services or that people may be subject to mistreatment is particularly concerning. Concerns about substandard care or mistreatment were more strongly held among trans and gender diverse participants (see Table 4a), and the lack of recognition of sexual or gender diversity by palliative care services has been previously commented upon (Aldredge & Conlon, 2012; Cartwright et al., 2010; Duffy & Healy, 2014). Bisexual people also often suggested care staff and services as well as concern of substandard care or mistreatment could be a barrier (see Table 4a).

It is reassuring to know that the majority of respondents who had experienced palliative care expressed this in a positive manner. Nevertheless, the findings indicate actions services can take to enhance their responsiveness to the needs of LGBTQ people. Staff training and building awareness of the health issues and needs of LGBTQ people were identified by several participants. This is a commonly mentioned strategy in the literature (Arthur, 2015; Barrett & Wholihan, 2016; Carabez & Scott, 2016; Cloyes et al., 2018; Reygan & D'Alton, 2013), which notes the importance of developing the LGBTQ‐sensitive knowledge of service providers. The review and update of service delivery have also been encouraged (Bristowe et al., 2018; Maingi et al., 2018; Stevens & Abrahm, 2019).

Services could review and improve their responsiveness by understanding how various components of appropriate palliative care identified in key local strategic documents have relevance for specific population groups.

### 4.4 Community‐level barriers and enablers

The lack of data about LGBT patients (Maingi et al., 2018) may mean that patients' partners, chosen families and/or next of kin may not be correctly identified by services, which may lead to partners or chosen family members potentially not having access to the patient facilitated, or potential conflict. It is important that services are responsive to diverse forms of kinship and chosen family, and improved data capture on sexuality, gender and people with intersex variations could particularly benefit palliative care. Targeted strategies to encourage advanced care planning among LGBT people have been suggested (Hughes & Cartwright, 2014, 2015), which could help ensure that people receive end of life and palliative care aligned to their wishes and needs.

By engaging with community(ies), for example, through community events, venues or social media, services might improve literacy and understanding of palliative care, and build trust in their capacity to meet the varied needs of the community(ies). Some LGBTQ community members and service providers lack knowledge of legal rights at the end of life (Carabez & Scott, 2016; Cartwright et al., 2018), suggesting a requirement for awareness‐raising.

It is important for palliative care information and promotion to represent the diversity of the community(ies), including cultural diversity. This information should aim to improve understanding of palliative care and provide reassurances of the inclusiveness of services. Engagement with community(ies) would be particularly important to ensure effective dissemination of palliative care information.

### 4.5 Society, policy and systems level barriers and enablers

Whilst some participants identified loneliness and isolation in the context of living in regional, rural and remote areas, other participants living in metropolitan NSW stated that loneliness and isolation could cause a barrier to accessing palliative care (Table 4a).

The increased levels of loneliness and social isolation raised by some community members have been previously identified in international research for older LGBT people (de Vries et al., 2019; Hughes, 2015), First Nations people who are trans living in remote areas (Kerry, 2015, 2017) and LGBT people from culturally and linguistically diverse backgrounds (Logie et al., 2016).

Some participants identified social isolation (from other community members) in the context of living in regional, rural and remote areas and to the increased conservatism that can exist outside of cities. The increased prevalence of homophobic attitudes and discrimination outside of cities has been noted (Morandini et al., 2015), often as a result of particular social features of regional areas (Ullman, 2015).

Whilst a few participants raised societal factors such as the introduction of marriage equality legislation, no clear themes in the enablers were apparent. The introduction of marriage equality in some countries may broaden societal understanding of the diversity of families.

### 4.6 Limitations

The use of social media to disseminate the community survey relied on participants' social networks to extend the reach of the survey within community(ies), therefore, these findings may not necessarily be generalisable to the whole LGBTQ population of NSW. A majority of participants lacked direct experience of palliative care, therefore the conclusions drawn from the participants' perspectives may not reflect directly on the appropriateness of palliative care services in NSW. The populations of interest included people who identify as having intersex variation(s), however, the researchers recruited only two people with intersex variation(s) who completed the survey, therefore the findings do not necessarily represent the views of this group. Further studies including this population warrant targeted approaches and materials, to ensure appropriate representation (Carpenter, 2012). It must also be acknowledged that the community survey did not include a question about Indigenous status.

### 4.7 Implications for practice

The literature from the past 10 years, relating to LGBTQ patient experiences of palliative care is particularly limited. Kemery (2021) provides a useful comparator of quality of end‐of‐life for LGBTQ and non‐LGBTQ individuals, and Hunt et al. (2019) provide the only exploration of we have found of experiences of LGBTQ people in low‐/middle‐income nations (in their case, Zimbabwe). Our research uses insights from LGBTQ people—with varied experience of palliative care—to form recommendations for more inclusive services. Our findings may help to raise awareness among workers providing palliative care of the perspectives and concerns of LGBTQ people. In addition, they may foster the provision of more effective, and appropriate palliative care for people of diverse sexualities and genders, including services delivered in other jurisdictions and comparable countries. This investigation aimed to articulate community views relating to palliative care services, however, the insights will be useful for generalist clinicians involved in delivering palliative care.

Trans and gender diverse individuals experience particular and unique challenges and obstacles in accessing health services more broadly (Grey & Janus, 2018; Rider et al., 2018). Palliative care services could consider how service provision can be enhanced to better support these individuals.

This research has strengthened understanding on views of LGBTQ people in NSW on ways to make palliative care more inclusive and appropriate for them. It can help policy makers and providers involved in the delivery of palliative care to make service‐level and system‐wide improvements to support these communities.

### 4.8 Implications for use of the SEM

Interrelations between factors presented in Table 5 is evident in themes such as ‘biological family issues as a source of tension’ (individual level) and ‘next of kin and decision making’ (community level). Tensions between patients and their biological family might influence decisions about next of kin, and vice versa. The SEM can assist those interested in improving palliative care for LGBTQ people, by providing a framework to understand multi‐factorial influences.

### 4.9 Implications for research

To our knowledge, this paper has revealed, for the first time how the SEM can be used to examine LGBTQ community members' perspectives on palliative care and how this model provides a useful framework to conceptualise multi‐factorial barriers and enablers to accessible and appropriate care. Opportunity exists for the SEM to be used to examine perspectives and care for other population groups. Future investigation into intersectionality of identities and palliative care would help to identify considerations for groups whose needs and preferences may be unrecognised.

Considerable diversity exists within the trans and gender diverse ‘umbrella’ group (Marshall et al., 2019; Zwickl et al., 2019), incorporating experiences (e.g., cis and trans) and identities (e.g., non‐binary). Participant numbers within subgroups in our study were not large enough to perform meaningful subgroup analysis. Future research should enquire into whether there are more variations in barriers, needs and enablers to palliative care for groups including cisgender, transgender and non‐binary people, using inclusive research approaches (ACON, 2021).

Palliative care literature about people with intersex variations is particularly lacking, and further targeted research about appropriate palliative care responses is recommended.

## AUTHOR CONTRIBUTIONS

NR, RP, LH and GR conceptualised the study. NR conducted data collection, and NR and IDS conducted data analysis. All authors—NR, RP, LH, GR, IDS, RI and ENS —contributed to data analysis and interpretation included in this paper. NR wrote the initial draft and led revisions to the paper. All authors contributed to revisions and agreed the final draft. NR and GR have ongoing roles in research translation for the findings.

## CONFLICT OF INTEREST

The authors have no conflicts of interest to declare.

## ETHICS STATEMENT

The study was approved by the University of New South Wales Human Research Ethics Committee (ref: HC200086). LGBTQ community organisational ethics approval was also provided by the ACON Research Ethics Review Committee (ref: 2020/09).

## Supporting information


Appendix S1

Appendix S2

Appendix S3

## Data Availability

Research data are not shared.
