# Supplementary material for: Lesbian, gay, bisexual, transgender and gender diverse and queer (LGBTQ) community members' perspectives on palliative care in New South Wales (NSW), Australia
Source: Health Soc Care Community. 2022 Sep 19;30(6):e5926–45. doi: 10.1111/hsc.14024 (PMC10087150; doi:10.1111/hsc.14024)
Supplement: Supplementary file 1 — Appendix S1 [file HSC-30-e5926-s001.pdf]

Appendix A: Community survey instrument

Research study: barriers and enablers to palliative care for people who are lesbian, gay, bisexual, transgender and gender diverse and/or people with intersex variations (LGBTI) in New South Wales (NSW)

LGBTI community survey on palliative care in NSW

You are invited to take part in this research study. It is being carried out by the University of New South Wales, in partnership with NSW Ministry of Health (UNSW HREC reference HC200086). The research study aims to help us understand the challenges some people who are lesbian, gay, bisexual, transgender and gender diverse and/or people with intersex variations (LGBTI) may experience with palliative care services towards the end of life. We would also like to know about what would help to improve these services for LGBTI people in NSW. You do not need to have experience or knowledge of palliative care services to take part in this survey.

We would like you to read the participant information statement before you start the survey (insert link).

Please read the following statements and click the option below:

- I am 18 years of age or older
- I understand I am being asked to provide consent to participate in this research study
- I have read the Participant Information Statement
- I provide my consent for the information collected about me to be used as described in section 7 of the Participant Information Statement
- I understand that if necessary, I can contact the research team to ask questions
- I freely agree to participate in this research study as described and understand that I am free to withdraw at any time during the study and withdrawal will not affect my relationship with any of the named organisations and/or research team members
- I understand that I can download a copy of this information statement and consent form from the link above
- I understand that if I would like to receive a copy of the study results via email or post, I can contact the research team
- I currently live in NSW.

If you agree to the conditions above, and would like to take part in the survey, please click 'I agree/continue' below

- I agree / continue (continue to A1)
- I do not agree and do not want to take part (go to end ‘thank you’ screen).

Section A: Introduction

Firstly, some questions to confirm that this survey is meant for you.

A1.	Do you consider yourself to be:	Single response required
	Lesbian, gay or homosexual	1
	Bisexual	2
	Straight or heterosexual	3
	Another identity (please specify)	90
	Prefer not to say	99

(question A1a only appears if people respond 'Another identity' to QA1.)

A1a	Open text field for 'Another identity'	Open response

A2.	Were you born with a variation of sex characteristics? (This is sometimes called 'intersex', 'DSD' or 'intersex variation' and includes people with variations in sexual anatomy, reproductive organs, hormonal patterns and chromosomal patterns that don't fit medical and social norms for female or male bodies)	Single response required
	Yes	1
	No	2
	Prefer not to say	99

A3.	Which of the following best describes your current gender identity?	Single response required
	Male	1
	Female	2
	Non-binary / gender fluid	3
	Different identity (please specify)	90
	Prefer not to say	99

(question A3a only appears if people respond 'Different identity' to QA3.)

A3a	Open text field for 'Different identity'	Open response

A4.	What was the sex recorded on your original birth certificate?	Single response required
	Male	1
	Female	2
	X/Indeterminate/Unspecified	3
	Other (please specify)	4
	I'm not sure	98
	Prefer not to say	99

A4.	This question is so we can compare people who live in metropolitan and country areas of NSW.<br>Do you live in a metropolitan or non-metropolitan part of NSW? (see map below for guidance)	Single response required
	Metropolitan (I.e. the Sydney, Newcastle and Wollongong areas marked in the map below)	1
	Non-metropolitan (I.e. another part of NSW)	2
	Prefer not to say	99

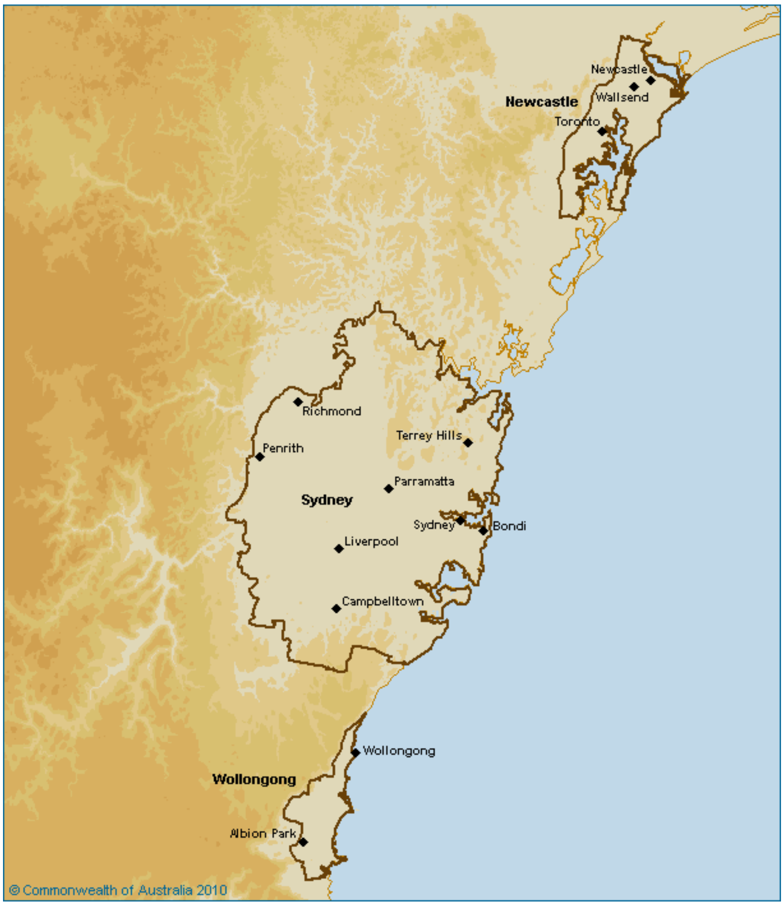

Image used from www.bom.gov.au under Creative Commons (CC) Attribution 3.0 license

Section B: awareness and opinions on palliative care services

Before the next questions, here are a few facts about palliative care, so we can make sure everyone completing this survey has the same understanding.

Palliative care aims to improve the quality of life of people with a life-limiting illness as they head towards the end of their life. It can also help their families or carers and others close to them. Palliative care can include help with people's physical, social, emotional and spiritual needs. It can be provided in various places, including at home, in hospital, in a hospice or a residential aged care facility. It can last weeks, months or even longer than a year.

The type of care that is available will depend on patients' and carer needs and local services available. Palliative care includes medical, nursing, allied health (e.g. physiotherapy, psychology, social work) and other services to manage changing health needs. This may include pain /symptom management, medication management, counselling and support services, providing patients with equipment, referrals to other services for personal care and home care services, respite care, volunteer services and bereavement support. It can be delivered by various people: doctors, nurses and allied health professionals, volunteers and carers. Together these people make up a 'palliative care team'.

In the following questions, the acronym LGBTI has been used in place of: 'people who are lesbian, gay, bisexual, transgender or gender diverse and/or people with intersex variations'.

Please take the time to read the following two short fictional examples of people receiving palliative care, to give you an idea of what people may experience.

"My partner, Alex was getting treated for bowel cancer for six months and then started to get a lot worse. The oncologist mentioned palliative care, which freaked me out a bit, because I thought that meant she only had a few days left. The oncologist sat down with us and explained what palliative care is, and made it clear that it is about trying to improve quality of life when someone has a serious illness that can't be cured.

They introduced me to our local palliative care team, who met with us, got to know Alex and I and helped us to organise little changes around the apartment, so Alex could stay as independent as possible.

The team helped us to get hold of a pressure relieving mattress and some sheepskin for Alex's chair. These helped her to be a lot more comfortable. They helped me to understand the medicines Alex was on, and how they may interact with supplements she was also taking. The team also taught us about some common symptoms for palliative patients, like pain, constipation, nausea, breathlessness and feeling tired. Alex hadn't exercised for a long time before the cancer, but the palliative care team encouraged her to go out for a walk a day. Alex improved a bit after we started these walks, and we were able to go on a short road trip – this had been Alex's dream for a long time.

Overall, Alex had support from the palliative care team for about 5 months. It was only in the last few days that Alex needed to go into hospital. The palliative care team really helped Alex to live as comfortable as possible right until the end."

Here is a different, fictional experience.

"My uncle was being treated for kidney failure and was in hospital for quite a few months before he died. The palliative care nurse would sit with him and listen to how he was going with his treatments and daily life. Uncle found that quite a relief, to have someone to talk to, as well as us family.

He spent loads of time hooked up to the dialysis machine, and he was sometimes in pain and couldn't sleep well. The team at the hospital changed his medication to try to make this pain better, and he seemed to sleep more easily after that.

A social worker saw him a couple of times in the hospital and when uncle mentioned he wanted to make peace with another family member, Aisha, they helped us to track Aisha down and arrange for

her to visit uncle the day before he died. This made a big difference, and helped things for us, as a family."

I can't say it was a pleasant experience overall, but the palliative care nurses did what they could to help him in his last days, and listen to him. They also helped reassure us because obviously it's a really stressful time."

B1.	Were you aware of palliative care before you started this survey?	Single response required
	I had not heard about palliative care before this survey	1
	I had heard about palliative care before, but did not really know what it is	2
	I had heard of palliative care before and understand what it is	3
	I prefer not to say	99

When you consider these next questions, even if you don't have experience of palliative care, you may want to draw on your experiences of other health services, and think how they may apply in palliative care.

B2	What do you think could be some of the difficulties with using palliative care services for LGBTI people?	Open response

B3	How could palliative care services make LGBTI people feel comfortable using them?	Open response

B4	How are the palliative care needs of the LGBTI community different to the rest of the population?	Open response

B5	How could NSW Health help LGBTI people to know what palliative care is and what services are available?	Open response

Features of palliative care services

B6.	Below are some statements about some features of palliative care services. Please think about how you would feel if you were to receive palliative care, and click the three that would be most important to you.
Order to be	My care (from the service) is based on my unique needs and preferences
	I am involved in decisions about my care
	I am not asked inappropriate questions about my body or my identity

rando-mised	Services are flexible in how they provide care
	I am treated with dignity and respect
	I am treated without prejudice or discrimination
	I am able to maintain my own sense of identity
	I am able to live the life I choose
	I can make informed choices
	My care is provided in a safe environment
	My family of choice/chosen carer are treated with dignity and respect

B7	As mentioned before, palliative care can be delivered in various places. If you needed to receive palliative care, what would be your preferred place to receive it (please click on the options and drag/move them to the order you prefer, where 1 is the option you would most prefer and 4 is the option you would least prefer)	Forced ranking from 1-4
Response options to be randomised	Home	1
	In hospital	2
	In a hospice (i.e. a facility that only provides specialist palliative care)	3
	In a residential aged care facility	4
	Don't know	98

B8	Please could you comment on why you chose this order?	Open response

Section C: use of palliative care services

C1.	Have you (or has someone close to you) used palliative care services? (select as many as apply)	Multiple responses allowed
	I have used these services for myself	1
	My current or ex partner has used these services within the past 10 years	2
	Someone else close to me (who also identifies as LGBTI) has used these services	3
	None of these options apply to me	8
	I prefer not to say	99

If C1 = 1 go to C2. (i.e. if respondent received pall care themselves)

If C1 = 2 or 3, go to C5 (i.e. if respondent's current/ex partner or LGBTI person close to them has received pall care)

If C1 does not equal 1, 2 or 3, or if C1 = 8 or 99, go to C7 (i.e. if no experience of pall care – either for respondent or other LGBTI ppl close to them)

C2	Overall, how did or does the palliative care service treat you?	Open response
C3	How did or does the palliative care service help you to maintain your own identity, or help you live the life you choose?	Open response

C4	‘Family of choice’ is used here to describe people close to you and supporting you – they may be biological family, friends, partners, or others important to you.<br>How were your family of choice or chosen carers treated by the palliative care service?	Open response

If C1 = 4, 5, 6 or 7 continue to C5 (i.e. if current/ex partner or another LGBTI person close to them has palliative care experience)

Otherwise, go to C7

C5	How does or did the palliative care service treat the person close to you?	Open response

C6	How does or did the palliative care service help the person close to you to maintain their own identity, or help them to live the life they chose?	Open response

C7	Are there any other comments you would like to make?	Open response

Section D: Demographics

These questions are not to identify you, but to help us to make sure we have a broad representation of experiences

D1	What is your age?	Single response required
	18-19 years old	1
	20-29 years old	2
	30-39 years old	3
	40-49 years old	4
	50-59 years old	5
	60-69 years old	6
	70-79 years old	7
	80+ years old	8
	I’d prefer not to say	99

D2	Do you speak a language other than English at home?	Single response required
	Yes – I speak a language other than English at home (please specify – write in required)	1
	No – I speak only English at home	2
	I'd prefer not to say	99

D3	In which country were you born?	Single response required
	Australia	1
	China	2
	England	3
	India	4
	New Zealand	5
	Philippines	6
	Other (please specify)	90
	I'd prefer not to say	99

D4	Do you identify as a person with a disability?	Single response required
	Yes	1
	No	2
	I'd prefer not to say	99

Thank you for sharing your experiences and opinions.

For more information about NSW Health palliative care services, please visit:
https://www.health.nsw.gov.au/palliativecare/Pages/default.aspx

If you feel upset after this survey, please contact one of the helpline numbers below to speak to somebody:

Q Life – 1800 184 527 (available 3pm-midnight, 7 days)

Lifeline- 13 11 14 (available 24 hours, 7 days)

The following organisations may be able to help you with information:

Palliative Care NSW- 8076 5600 or email: info@palliativecarensw.org.au

Intersex Peer Support Australia (IPSA) - 0478 537 739 or email info@isupport.org.au
