# Supplementary material for: Lesbian, gay, bisexual, transgender and gender diverse and queer (LGBTQ) community members' perspectives on palliative care in New South Wales (NSW), Australia
Source: Health Soc Care Community. 2022 Sep 19;30(6):e5926–45. doi: 10.1111/hsc.14024 (PMC10087150; doi:10.1111/hsc.14024)
Supplement: Supplementary file 2 — Appendix S2 [file HSC-30-e5926-s003.pdf]

Appendix B: Discussion guide for follow up interviews

Research study: barriers and enablers to palliative care for people who are lesbian, gay, bisexual, transgender and gender diverse and/or people with intersex variations (LGBTI) in New South Wales (NSW)

Discussion guide for follow up interviews with LGBTI community members

1. **Introduction**
   I'm on a research team that's conducting a study to help us understand the challenges some people who are lesbian, gay, bisexual, transgender and gender diverse and/or people with intersex variations (LGBTI) may experience with palliative care services towards the end of life. We would also like to know about what would help to improve these services for LGBTI people in NSW.

2. **Invitation**
   Thank you for completing the online survey, and for getting in touch with me. I emailed you a participant information statement for this interview, can you let me know if you have read this?
   - If they have not received it, check email address with participant, and email it to them again, and re-schedule interview (e.g. for following day).
   - If they have received it, but not read it yet. Ask them to read the PIS, and reschedule the interview.
   - If they have read it, proceed with the following information.

   I would like to invite you to participate in this follow up phone interview, as part of the same research study. The questions will build on the survey questions, but we won't match your answers from today with your survey responses because the survey was anonymous. Before we go any further I need to let you know that participation in this research study is voluntary. If you do not want to take part, you do not have to. Are you happy for me to provide you with further information on the research study?
   - If no, thank the participant for their time and end the consent process.
   - If yes, proceed with the following information.

3. **Description of participation**
   If you decide to take part in this part of the research study, I will ask you to answer a few questions building on from the survey, about what palliative care means for you, what the needs are for the LGBTI community, and any experiences you may have had of palliative care services for yourself or others close to you. It should take approximately 30 to 45 minutes to complete. We don't expect the interview to cause any harm or discomfort, however if you experience feelings of distress as a result of participation in this study you can let me know and we will provide you with assistance. Just to ensure that I understand what you have to say correctly I would like to record this interview. But if you don't want to be recorded, I can make notes instead.

4. **Data storage and use**
   During this interview, I will collect information from you relevant to this research study. Your data will be kept for a minimum of seven years after we publish the study findings, after the project's completion. Your information will only be used for this particular research study.
   Any information we collect from you will be stored and presented in research publications and in conference presentations in a way that will not identify you.

5. **Withdrawal from the research**
If you decide to leave the research study, we will not collect additional information from you. Any identifiable information about you will be withdrawn from the research project, however since the original survey was anonymous, we will not be able to withdraw that information from the research project. Your decision not to participate or to withdraw from the study will not affect your relationship with UNSW Australia or NSW Health.

6. **Questions**
Do you have any questions in regards to the information that I have provided?
    - If yes, answer any questions the participant may have
    - If no, continue to collect consent.

If you would like, I will send you an email containing the details of the person for you to contact if you have any questions or complaints about the research study.

7. **Consent**
Now that I have explained what your involvement in the research study requires, are you happy to provide your consent to participate in the study?
    - If no, thank the participant for their time and end the consent process.
    - If yes, record the time and date the verbal consent was collected from the participant, and ask the following:
        - would you like a copy of the participant information statement (what I have just read out to you) emailed to you?;
        - do you agree to me using an audio recorder to recording this interview
            - If yes, start audio recorded and commence data collection
            - If no, let participant know I will take notes instead, and start data collection.

(Reinforce that I can skip any questions they don't feel happy answering. Check if they have any questions before interview starts.)

Questions

1. To start off, can you briefly introduce yourself (just your first name please), and let me know what palliative care means to you in general?

    Prompts: and do you have experience of palliative care for yourself or another LGBTI person close to you?

Question piping: for people who have experienced palliative care (either for themselves, a partner or another LGBTI person close to them, skip questions 2-4 and go to question 5. For people without this experience, go to question 2.

2. Even though you don't have experience of palliative care, when thinking about these next questions, you can draw on your experiences of other health services, and think how they may apply in palliative care.
I'd like to hear from you about what would 'appropriate' palliative care be for the LGBTI community (spell out)?

    Prompts: how could palliative care services address:
    - involving LGBTI people in decisions about their care
    - treating LGBTI people with dignity and respect

- treating LGBTI people without prejudice or discrimination
- treating LGBTI people's family of choice/chosen carer with dignity and respect.

3. Now I'd like you to let me know what would 'accessible' palliative care be for the LGBTI community?

Prompts: What makes you say that?
What would make it more difficult for LGBTI people to access palliative care services?
What do palliative care services need to do to make their services more accessible for the LGBTI community?

4. Would you say that the palliative care needs of LGBTI people are different to or the same as the rest of the population?

Prompts: What makes you say that?
(if they say the needs are different) How are their needs different?
What do palliative care services need to do to meet these needs for the LGBTI community?

5. If you were to need palliative care, how would you expect to be treated by the palliative care team?

Prompts: What makes you say that?
We don't have to talk about this if you don't feel comfortable, but have you had previous experiences from health services like this?
How would you expect the palliative care workers to treat those close to you?

Questions 6-13 are for people who have experienced palliative care themselves or a partner / ex partner or if another LGBTI person close to them has experienced palliative care. For all others, go straight to Q14.

6. Thanks for answering those general questions about palliative care. If it's ok with you, I'd like to now ask you some questions about your (or other person's) experience. Could you please let me know how long ago you (or someone close to you) were involved with a palliative care service?

Prompts: Who was receiving the palliative care in your situation? *(tailor subsequent questions to first or second person, accordingly)*

7. And where was the palliative care service being provided? (you don't need to let me know the name, if it was a health facility)

Prompts: in hospital? In residential aged care? At home? Hospice? Another place? Multiple places at different times?

8. And if you can remember, how did you/they hear about the palliative care service?

Prompts: How easy was it to start receiving this service?
Did someone or a service refer you/them?
How did the contact come about?
What are the challenges around accessing or receiving palliative care in your local area?

9. In the survey you filled in, we asked you broadly about how the palliative care service treated you/them. I now have some more specific questions about that.

Prompts: How safe did the palliative care service make you/them feel?
How comfortable did the service make you feel?
What did the palliative care service do to treat you/them with dignity and respect?

10. How well did the palliative care service meet your/their specific needs? And would you say they met you/their preferences?
Prompt: Can you think of an example of this?

11. How much were you/xxx involved in decisions about care and treatment?

Prompts: Was this level of involvement and decision making as much as you/they wanted?
How did the service support you/xxx to make choices?
Did the service provide any information to make these choices easier?

12. Thank you for your answers so far. I'd like to move on to your thoughts in general on palliative care for the LGBTI community. Could you let me know what would 'accessible' palliative care be for the LGBTI community?

Prompts: What makes you say that?
What would make it more difficult for LGBTI people to access palliative care services?
What do palliative care services need to do to make their services more accessible for the LGBTI community?

13. Would you say that the palliative care needs of LGBTI people are different to or the same as the rest of the population?

Prompts: What makes you say that?
(if they say the needs are different) How are their needs different?
What do palliative care services need to do to meet these needs for the LGBTI community?

General questions about palliative care for all interviewees

14. In the survey responses we received, many people suggested ways of letting LGBTI people know what palliative care is and the services that are available. What do you think should be the main message NSW Health should be saying about palliative care to LGBTI people in NSW?

15. Thank you for your answer so far. Is there anything else you'd like to add?

16. We're nearly at the end now. To help us identify how varied the people we are interviewing are, these are a few more questions about you (we won't use these to identify you in the research):

    a. LGBTI status (make it clear they don't have to answer any questions they don't want to):
        i) Do you consider yourself to be lesbian/gay/homosexual; straight/heterosexual; bisexual or another identity?
        ii) Were you born with a variation of sex characteristics (this is sometimes called 'intersex', 'DSD', 'intersex variation', and includes people with variations in sexual anatomy, reproductive organs, hormonal or chromosomal patterns that don't fit medical or social norms for male and female bodies)
        iii) Which of the following best describes your current gender identity: female; male; non binary, gender fluid or another identity?
        iv) Which was the sex recorded on your original birth certificate (male, female, X/indeterminate/unspecified, or something else)?.
    b. Which age range would you be? (18-19 years, or in your 20s, 30s, 40s, 50s, 60s, 70s or 80s and above?)
    c. Do you speak a language other than English at home?
    d. Could you please let me know your country of birth?
    e. Do you identify as a person with a disability?
    f. Do you live in metropolitan NSW or not? (show them map, if required)

End of interview – switch recorder off.

Thank participant for their time and check if they have any final questions for me.

If they are distressed, talk through with them, and follow process for distressed participants.

If not distressed, ask them if they would like any contact numbers for Lifeline or Q Life telephone counselling, in case they feel they would like to speak with someone later.

- Lifeline (24 hours)131114
- Q Life (3pm to midnight) 1800 184 527

Let the participant know that they may also wish to contact one of the following organisations:

- Palliative Care Australia - 8076 5600 (available 9am to 5pm, Monday to Friday)
- Intersex Peer Support Australia (IPSA) - 0478 537 739
