# Supplementary material for: Lesbian, gay, bisexual, transgender and gender diverse and queer (LGBTQ) community members' perspectives on palliative care in New South Wales (NSW), Australia
Source: Health Soc Care Community. 2022 Sep 19;30(6):e5926–45. doi: 10.1111/hsc.14024 (PMC10087150; doi:10.1111/hsc.14024)
Supplement: Supplementary file 3 — Appendix S3 [file HSC-30-e5926-s002.pdf]

Supporting Information: Glossary of terms and acronyms

(adapted from the NSW Health LGBTIQ Health Strategy, available at: https://www.health.nsw.gov.au/lgbtiq-health/Pages/default.aspx)

Terminology to describe sexualities, gender identities and intersex variations can change over time. Further information and definitions can be found at https://aifs.gov.au/cfca/publications/lgbtiq-communities and https://www.transhub.org.au/

Bodies

Sexual characteristics: physical parts of the body that are related to body development/regulation and reproductive systems. Primary sex characteristics are gonads, chromosomes, genitals and hormones.

People with intersex variations: people who are born with anatomical, chromosomal and hormonal characteristics that are different from medical and conventional understandings of female and male bodies. The term 'intersex' incorporates a wide range of physical variations and conditions. There are currently at least 40 relevant clinical entities known.

Endosex: used to describe people who are not intersex.

Gender

Gender: one's sense of whether they are a man, woman, non-binary, agender, genderqueer, genderfluid, or a combination of one or more of these definitions.

Gender experience: describes the relationship between a person's gender, and the gender they were presumed at birth.

Binary: binary genders are male and female, and non-binary genders are any genders that are not just male or female or aren't male or female at all.

Non-binary: an umbrella term for any number of gender identities that sit within, outside of, across or between the spectrum of the male and female binary. A non-binary person might identify as gender fluid, trans masculine, trans feminine, agender, bigender.

Transgender (trans) and gender diverse: these are inclusive umbrella terms that describe people whose gender is different to what was presumed for them at birth. Transgender people may position 'being trans' as a history or experience, rather than an identity, and consider their gender identity as simply being female, male or a non-binary identity.

Cisgender/Cis: a term used to describe people who identify their gender as the same as what was presumed for them at birth (male or female). 'Cis' is a Latin term meaning 'on the same side as'.

*Sistergirl/Brotherbo*y: terms may be used to refer to Aboriginal people who identify as gender diverse within some Aboriginal communities.

Affirmation: the process of socially, medically, legally or otherwise affirming a person's gender when it does not align to their sex assigned at birth.

Sexualities

Sexuality: describes a person's sexual, romantic, spiritual, or emotional attraction to other people

Lesbian: a woman who is attracted to women.

Gay: a man who is attracted to men.

Bisexual: a person who is attracted to people of their own and other genders.

Pansexual: a person who is attracted to any person

Queer: a range of non-normative genders and sexualities. Although once used as a derogatory term, the term queer now also encapsulates political ideas of resistance to heteronormativity and homonormativity and is sometimes used as an umbrella term to describe the full range of LGBTIQ+ identities.

Heterosexual: a person who is only attracted to people with a different gender to their own

Asexual/ace: a person with a lack or rarity of sexual attraction

This is not a comprehensive list. Definitions have been adapted from the following resources, where further information and definitions can be found: https://aifs.gov.au/cfca/publications/lgbtiq-communities and https://www.transhub.org.au/101/what-is-trans

Societal attitudes

Homophobia, biphobia, transphobia: negative attitudes, beliefs, prejudice, discrimination and actions towards people of diverse sexualities and to transgender and gender diverse people.

Heteronormative: a world view that promotes heterosexuality as the normal or preferred sexual orientation.

Cisgenderism: the belief that gender identity is determined at birth and is a fixed and innate identity that is based on sex characteristics (or 'biology') and that only binary (male or female) identities are valid and real.

Cisnormative: a world view that promotes cisgender people as the norm

Other terms used in this article

Chosen family: nonbiological kinship bonds, whether legally recognised or not, deliberately chosen for the purpose of mutual support and love

Metropolitan: for the purpose of this study, metropolitan NSW was classified as within the Sydney, Wollongong or Newcastle areas of NSW

Non-metropolitan: for the purpose of this study, metropolitan NSW was classified as areas of NSW other than Sydney, Wollongong or Newcastle.

Reference:

NSW Ministry of Health. (2022) *NSW LGBTIQ+ Health Strategy 2022-2027*. Available online: https://www.health.nsw.gov.au/lgbtiq-health/Publications/lgbtiq-health-strategy.pdf
